# The impact of serum lipids on risk for microangiopathy in patients with type 2 diabetes mellitus

**DOI:** 10.1186/1475-2840-11-109

**Published:** 2012-09-14

**Authors:** Peter P Toth, Robert J Simko, Swetha Rao Palli, Dawn Koselleck, Ralph A Quimbo, Mark J Cziraky

**Affiliations:** 1 CGH Medical Center, 101 east Miller Rd., Sterling, IL, 61081, USA; 2 University of Illinois College of Medicine, Peoria, IL, USA; 3 Abbott Laboratories, Abbott Park, IL, USA; 4 HealthCore, Inc., Wilmington, DE, USA

**Keywords:** Lipid subfractions, ADA treatment goals, Diabetes, Microvascular complications, Retinopathy, Neuropathy, Nephropathy

## Abstract

**Background:**

Few large-scale, real-world studies have assessed the relative associations of lipid fractions with diabetic microvascular events. The main objective of this study was to evaluate the association of the lipid profile components, high density lipoprotein cholesterol (HDL-C), low density lipoprotein cholesterol (LDL-C), triglycerides (TG), and non-high density lipoprotein cholesterol (non-HDL-C) with microvascular complications (MVCs) in type 2 diabetes mellitus (T2DM) patients.

**Methods:**

This observational cohort study queried the HealthCore Integrated Research Database (HIRD^SM^) for newly-diagnosed (Index Date) 18-64-year-old patients with diabetes mellitus between 01/01/2005-06/30/2010. Inclusion required ≥12 months pre-index continuous health plan eligibility and ≥1 pre-index lipid profile result. Patients with polycystic ovary syndrome and prior MVCs were excluded. Incident complications were defined as the earliest occurrence of diabetic retinopathy, peripheral neuropathy, and/or nephropathy post-index. Cox proportional models and Kaplan-Meier (KM) curves were used to evaluate associations among variables.

**Results:**

Of the patients (N = 72,267), 50.05 % achieved HDL-C, 64.28 % LDL-C, 59.82 % TG, and 56.79 % non-HDL-C American Diabetes Association goals at baseline. During follow-up (mean, 21.74 months), there were 5.21 microvascular events per 1,000 patient-months. A 1-mg/dL increase in HDL-C was associated with 1 % decrease in any MVC risk (*P* < .0001), but for LDL-C, TG, and non-HDL-C, 1-mg/dL increase resulted in increases of 0.2 % (*P* < .0001), 0.1 % (*P* < 0.001) and 0.3 % (*P* < 0.001) in MVC risk. Patients achieving HDL-C goals had a 11 % lower risk of MVC versus non-achievers (RR 0.895, [95 % CI, 0.852-0.941], *P* < .0001). Similarly, TG goal attainment was associated with a lowered risk for any MVC (RR 0.849, [95 % CI, 0.808-0.892], *P* < .0001). Evaluation of KM survival curves demonstrated no significant difference in the risk of MVCs between patients achieving vs. not achieving LDL-C goals, but did demonstrate a difference in MVC risk between patients achieving vs. not achieving non-HDL-C goals.

**Conclusion:**

This study demonstrates significant independent associations among lipid fractions and risk for microangiopathy. These findings suggest that attaining established ADA goals for HDL-C, TG, and non-HDL-C may reduce risk for microvascular events among patients with diabetes.

## Background

An estimated 25.8 million people, or 8.3 % of the US population, were affected by diabetes mellitus in 2010, with 90 %-95 % afflicted with type 2 (T2DM). The American Diabetes Association (ADA) and American Heart Association (AHA) attribute substantial patient morbidity and mortality to T2DM [1]. Furthermore, T2DM is associated with multiple microvascular complications (MVCs) including retinopathy, neuropathy, and nephropathy, all of which contribute to diabetes-associated morbidity and mortality [2,3]. From 2005–2008, 4.2 million patients with diabetes had diabetic retinopathy, 655,000 of whom developed serious vision loss [4] for a total annual cost of $492.98 million [5]. Diabetic neuropathy affects an estimated 12 %-50 % of those with diabetes, with total annual costs estimated at $10.1 billion. As many as 19 % of T2DM patients have nephropathy and end-stage renal disease, with annual medical costs in the US amounting to about $15 billion [6].

Elevated serum levels of low-density lipoprotein cholesterol (LDL-C) and triglycerides (TG) and low levels of high-density lipoprotein cholesterol (HDL-C) are strongly associated with increased risk for macrovascular events (e.g., myocardial infarction, ischemic stroke, and coronary mortality) among patients with T2DM [3,7,8]. However, no consensus exists on possible mechanisms linking these individual lipid subfractions to MVCs. A systematic review of studies assessing associations between lipid subfractions and MVCs noted that dyslipidemia may cause or worsen complications [3]. A European study reported significant associations between elevated levels of total cholesterol (TC) and lower levels of HDL-C with increasing severity of diabetic retinopathy; there was, however, no apparent association between serum TG and retinopathy [9]. Another study found high levels of TC and TG associated with diabetic nephropathy and declining kidney function [10]. The relationship between abnormal lipid subfractions and diabetic neuropathy is relatively unexplored, with only one study showing a small degree of association [11]. Most studies seeking to establish associations between dyslipidemia and microvascular events were of short duration with relatively small sample sizes, rendering them underpowered and with limited generalizability [3]. Nevertheless, available evidence indicates abnormalities among lipid subfractions exacerbate the risk of microvascular events [9].

To date, few studies have investigated the relative magnitude of association between individual lipid subfractions and diabetes-related MVCs. Furthermore, comprehensive literature reviews have not revealed any large-scale real-world studies that assessed the relative associations of standard lipid fractions with diabetic MVCs. In recognition of that gap, this study accessed data from a managed care database to evaluate associations between the levels of lipid subfractions (LDL-C, HDL-C, TG, and non-HDL-C) and the incidence of diabetic retinopathy, peripheral neuropathy, and nephropathy. The impact on risk for MVCs following attainment of ADA targets for specific components of the lipid profile was also quantified [12].

## Methods

### Data source and study design

This observational cohort study utilized integrated medical, pharmacy, and laboratory result data from the HealthCore Integrated Research Database^SM^ (HIRD^SM^) between 01/01/04-06/30/2010. The HIRD^SM^ contains administrative claims data for more than 35 million Americans covered by 14 geographically dispersed commercial US healthcare plans. All information utilized in this retrospective, longitudinal analysis was managed in strict compliance with the Health Insurance Portability and Accountability Act (HIPAA) of 1996. Patient confidentiality and anonymity was preserved via de-identification throughout the study.

### Inclusion criteria

Patients newly diagnosed with T2DM (index date) involving a medical claim with an *International Classification of Diseases*, Ninth Revision, Clinical Modification (ICD-9-CM) diagnosis code for 250.x0 or 250.x2 or a pharmacy claim with a GPI for an antidiabetic agent between 01/01/2005 and 06/30/2010 were identified. The index date was the earliest point of onset of T2DM. To be included, patients aged between 18-to-64 years were required to have ≥12 months of continuous health plan eligibility preceding the index date and ≥1 full lipid panel during the 12-month pre-index date period, the most current of which was defined as the baseline lipid panel.

### Exclusion criteria

Patients with a history of T2DM or any pre-index medical claim for diabetic retinopathy, neuropathy, or reduced kidney function were excluded. Also excluded were patients with a medical claim for polycystic ovary syndrome (ICD-9-CM: 256.4x) any time during the study period.

### Definition of follow-up

Follow-up was defined as the period between the index date and the earliest of the following: end of continuous health plan eligibility, end of the available data stream (06/30/2010), or a censoring event. For each outcome of interest, data were censored at the earliest occurrence of the event of interest, when patients reached 65 years of age, or a death was recorded in the Social Security Death Index Master File. Adjustments in the amount of person-time contributed during follow-up were made whenever applicable to account for follow-up periods of varying durations.

### MVCs

The MVCs of interest were diabetic neuropathy, retinopathy, and nephropathy identified during any inpatient, outpatient, or emergency room visit using ICD-9-CM diagnoses and procedure codes, Current Procedural Terminology (CPT) codes, and revenue service codes from patients’ medical claims. Nonspecific diagnoses of neuropathy and nephropathy were considered to be T2DM-attributable MVCs provided a T2DM diagnosis code was recorded on the same medical claim date. In addition, diabetic nephropathy was also identified using glomerular filtration rates (eGFR) estimated from the serum creatinine laboratory values. An abbreviated Modification of Diet and Renal Disease (MDRD) equation was used to calculate eGFR since information on race was lacking [13]. An eGFR of <60 mL/min was defined as reduced kidney function, although this may not necessarily be attributable to diabetes. As a result, any eGFR-identified overt nephropathy was only classified as diabetic nephropathy if there was no prior medical claim for nonspecific nephropathy.

### Lipid subfraction goals

Results from the last full lipid panel measurement before the end of the follow-up period was used to evaluate the relative association between each lipid subfraction and the events of interest. The target level for each lipid fraction was defined on the basis of ADA treatment guidelines for people with diabetes: LDL-C <100 mg/dL, HDL-C >40 mg/dL for men and >50 mg/dL for women, TG <150 mg/dL, and non-HDL-C <130 mg/dL [12]. Assessment of lipid goals as of the baseline lipid panel (i.e., preceding the onset of T2DM), were applied according to the National Cholesterol Education Program Adult Treatment Panel III (NCEP ATP III) Guidelines for nondiabetic patients [13]. Depending on their risk category — primary (2+ risk factors), secondary (cardiovascular heart disease [CHD] or CHD risk equivalents), or non-determinant (0–1 risk factors) based on information available from their start date of continuous eligibility until the day of the lipid panel measurement — the following rules for goal attainment were applied: LDL-C <130 mg/dL and <100 mg/dL for patients at primary and secondary risk, respectively; HDL-C >40 mg/dL for men and >50 mg/dL for women; TG <150 mg/dL for women (based on AHA guidelines for women [14]) and <200 mg/dL for men; and non-HDL-C <160 mg/dL and <130 mg/dL for patients at primary and secondary risk, respectively. For patients with an indeterminate risk status, baseline LDL-C and non-HDL-C goal attainment could not be ascertained.

### Patient characteristics

Demographic characteristics included age, gender, geographic location, and type of health insurance coverage on index date. Baseline comorbidities and the Deyo-Charlson Comorbidity Index were evaluated during the 12-month pre-index period. The most recent lipid subfractions of interest (LDL-C, HDL-C, TG, and non-HDL-C) prior to the index date were used to describe the baseline lipid panel.

Medication utilization patterns were assessed for antiglycemic, antihypertensive, and lipid-altering therapies at baseline and follow-up. Exposure to both antidiabetic and antihypertensive agents (angiotensin-converting enzyme [ACE] inhibitors and angiotensin receptor blockers [ARBs]) during follow-up was measured based on the proportion of days covered (PDC) [15,16]. Exposure to lipid-altering therapies, such as statins, niacin, and fibrates (fenofibrate and gemfibrozil), as of the latest available full lipid laboratory panel prior to the end of follow-up. Was evaluated to adjust for potential changes in lipid values between the last observed lipid panel and the end of follow-up.

## Statistical analyses

### Descriptive statistics

Continuous characteristics were represented using means and standard deviations, while categorical variables were evaluated as percentages. All right-censored continuous outcomes were compared by means of unadjusted Cox proportional hazards models and the log rank test. Kaplan-Meier (KM) curves were constructed to evaluate the risk of MVCs between patients achieving and not achieving goals for each lipid subfraction.

### Multivariate analysis

Four Cox proportional hazards models were developed to determine the relative significance of lipid subfraction levels and goal attainment on the risk of MVCs. Additional covariates included age, gender, state of residence, health plan, and physician type. Also included were pre-index health conditions (hypertension, obesity, heart failure, metabolic syndrome, renal disease, liver disease, ischemic heart disease, peripheral vascular disease, and depression) and PDC during follow-up for antihypertensives (ACEs and ARBs) and lipid-altering therapies (statins, niacin, ezetimibe, and fibrates).

### Sensitivity analysis

Additional univariate assessments were performed to determine if the observed associations between TG/HDL and MVCs remained unchanged or attenuated once TG was adjusted for HDL-C or vice versa by computing the variance inflation factor (VIF) and condition index. A value of 10 and 30 respectively were set apriori to indicate a potential issue of multicollinearity. In order to further investigate the relative importance of the management of other lipid measures beyond LDL-C alone with respect to MVC risk, the risk of MVCs among patients achieving LDL-C goals only vs. those simultaneously achieving HDL-C and LDL-C goals was evaluated. Similar comparisons were conducted between those achieving LDL-C goal only vs. those simultaneously achieving dual goals of LDL-C and TG, HDL-C and TG and non-HDL-C goals respectively. Furthermore, the above analyses were replicated among those who did not attain LDL-C treatment goals to evaluate the impact of HDL-C, TG, and Non-HDL-C goal achievement in the absence of LDL-C goal achievement.

## Results

### Demographic and clinical characteristics at baseline

Of the patients (N = 72,267) in this study, 48.7 % were females, and the overall mean (±SD) age was 49.91 (±9.44) years (Table 1). At baseline, the mean (±SD) lipid panel values for all patients were 115.71 (±35.65) for LDL-C, 47.28 (±13.90) for HDL-C, 186.69 (±176.52) for TG, and 150.61 (±43.53) for non-HDL-C. Overall, 50.05 % of the patients attained their HDL-C goals, while 59.82 % reached their TG goals at baseline. Based on a subsample of 37,118 patients for whom CHD risk status and corresponding LDL-C and non-HDL-C goals could be determined, 64.28 % of the patients attained their LDL-C goals, while 56.79 % reached their non-HDL-C goals at baseline.

**Table 1:** Baseline Characteristics of Patients in the Study

**Characteristics**	**Patients N =72,267**	
	**n**	**%**
**Female**	35,192	48.70
**Age at index** (years)**,** mean ± SD	49.91	± 9.44
**Geographic Region**		
West	21,098	29.19
South	19,727	27.30
Northeast	15,756	21.80
Midwest	11,231	15.54
Unknown	4,455	6.16
**Health Plan Type**		
HMO	35,017	48.46
PPO	31,556	43.67
POS	2,347	3.29
FFS	65	0.09
Other	3,255	4.50
**Index year**		
2005-06	21,929	30.34
2007-08	30,767	42.57
2009-10	19,571	27.08
**Physician specialty**		
General family practice	22,927	31.34
Internal medicine	17,817	24.65
Endocrinology	1,963	2.72
Cardiology	800	1.11
Others/unknown	28,760	39.80
**Lipid Subfraction**^**1**^		
LDL-C	115.71	±35.65
Goal attainment^2^	23,858	64.28
HDL-C	47.28	±13.90
Goal attainment	36,171	50.05
TG	186.69	±176.52
Goal attainment	43,230	59.82
nonHDL-C	150.61	±43.53
Goal attainment^2^	21,081	56.79
**Comorbidities**		
Ischemic heart disease	66,428	91.92
Hypertension	42,605	58.95
Obesity	4,912	6.80
Depression	3,947	5.46
Heart failure	2,511	3.47
Cerebrovascular disease	2,023	2.80
Metabolic syndrome	1,940	2.68
Liver disease	1,932	2.67
Peripheral vascular disease	1,554	2.15
Renal disease^3^	394	0.55
Schizophrenia	68	0.09
DCI score, mean ± SD	0.34	±0.9
**Medication Use**^**4**^		
***Antihypertensive Medications***	14,387	19.91
ACE Inhibitors	10,444	14.45
ARBs	4,515	6.25
***Lipid Altering Therapies***		
Statin		
Low Potency	2,422	3.35
Medium Potency	9,840	13.62
High Potency	12,851	17.78
Niacin	1,668	2.31
Ezetimibe	3,827	5.30
Fibrate		
Fenofibrate	3,211	4.44
Gemfibrozil	1,254	1.74
**Days of Study Follow-up**^**5**^**, mean ± SD**	652.58	±484.18

^1^ The lipid panel values are derived from the most recent lab assessment results prior to the index date.

^2^ For LDL-C and non-HDL-C: Goal attainment based on subsample of 37,118 patients since 35,149 patients had no discernible risk at baseline.

^3^ Pre-index renal disease includes acute renal failure, hypertensive kidney disease, and glomerulonephritis.

^4^ n and % of patients with any fill for medication of interest.

^5^ Number of days from index date to end of study follow up (defined as the end of earliest occurrence of event of interest, death, patient becoming 65 years or older, end of health plan eligibility, or 6/30/2010).

LDL-C: low-density lipoprotein cholesterol; HDL-C: high-density lipoprotein cholesterol; TG: triglycerides; ACE: angiotensin-converting enzyme; ARB: angiotensin receptor blockers; HMO: health maintenance organization; PPO: patient preferred organization; POS: point of service; FFS: fee for service.

### Follow-up medication utilization

Assessment of medication utilization during follow-up revealed no significant differences between any medication class (i.e., antiglycemic, antihypertensive, and lipid-altering drugs) irrespective of whether patients had or did not have an MVC event for any of the lipid subfractions.

### Follow-up MVC incidence rate

On the basis of 1000 patient-months, the incidence rate for any microvascular event was 5.21 (95 % CI, 5.09-5.33). Incidence rates were 1.44 (95 % CI, 1.38-1.50) for diabetic retinopathy; 1.26 (95 % CI, 1.20-1.32) for diabetic neuropathy; and 2.61 (95 % CI, 2.53-2.69) for diabetic nephropathy.

### Follow-up Lipid Subfractions

As of the last full lipid panel measurement during follow-up, the mean HDL-C, TG, and non-HDL-C values for patients who experienced any MVC event were significantly different compared with patients with no events (*P* < .0001; Table 2). The mean HDL-C values in the total population was 48.02 (±14.13); for patients with an observed MVC event, the mean HDL-C was 46.72 (±14.04); and for patients without events, 48.16 (±14.13, *P* < .0001). The mean LDL-C values for patients who experienced MVC events versus those with no event were not significantly different.

**Table 2:** Last Lipid Panel Results Prior to Any Microvascular Event or Censorship

**Lipid Characteristics**	**All Patients**		**Event**^**1**^		**No Event**^**2**^		***P *- Value**^**3**^
	**N = 72,267**		**n = 7,271**		**n = 64,996**		
**# Days from Last Lipid Panel to Any Microvascular Event/Censorship: mean ± SD**	346.21	±351.43	148.40	±223.94	368.34	±356.15	<.0001
**Lipid Panel Values: mean ± SD**							
LDL-C	109.08	±34.35	108.33	±36.47	109.16	±34.1	.0513
HDL-C	48.02	±14.13	46.72	±14.04	48.16	±14.13	<.0001
Triglycerides	163.35	±122	186.39	±186.41	160.77	±112.23	<.0001
Non-HDL-C	140.55	±40.25	143.19	±45.9	140.26	±39.56	<.0001
**Lipid Goal Attainment Results: n, row%**							
LDL-C	30,395	42.06 %	3,185	43.80 %	27,210	41.86 %	.0015
HDL-C	37,946	52.51 %	3,527	48.51 %	34,419	52.96 %	<.0001
Triglycerides	43,577	60.30 %	3,948	54.30 %	39,629	60.97 %	<.0001
Non-HDL-C	30,737	42.53 %	3,032	41.70 %	27,705	42.63 %	.1299

^1^For those patients who experienced any corresponding microvascular event of interest (diabetic retinopathy/nephropathy/neuropathy), mean values of the last laboratory assessment prior to the first event are presented.

^2^For those patients who did not experience the microvascular event of interest (diabetic retinopathy/nephropathy/neuropathy), mean values of the last laboratory assessment prior to censorship (death, age >64 years, end of plan eligibility, or data period) are presented.

^3^*P*-value between the event and no event groups is significant at < .05.

LDL-C: low-density lipoprotein cholesterol; HDL-C: high-density lipoprotein cholesterol; TG: triglycerides.

LDL-C goal attainment occurred more frequently among patients with events (43.8 %) versus those who had no event (41.86 %, *P* = .0015). Patients who experienced an MVC event were less likely to have attained their HDL-C and TG goals (48.51 % and 54.30 %, respectively) compared with patients with no event (52.96 % and 60.97 %, for HDL-C and TG, respectively, *P* < .001). There was no significant difference in the proportion of patients achieving non-HDL-C goals between patients with MVC events (41.70 %) compared with those with no events (42.63 %, *P* = .1299).

### Univariate and multivariate comparisons between lipid subfractions and MVCs

MVC incidence rates per 1000 patient-months were evaluated for each lipid subfraction based on the laboratory values from the last tests before the first event or censorship.

#### LDL-C

**Figure 1:**
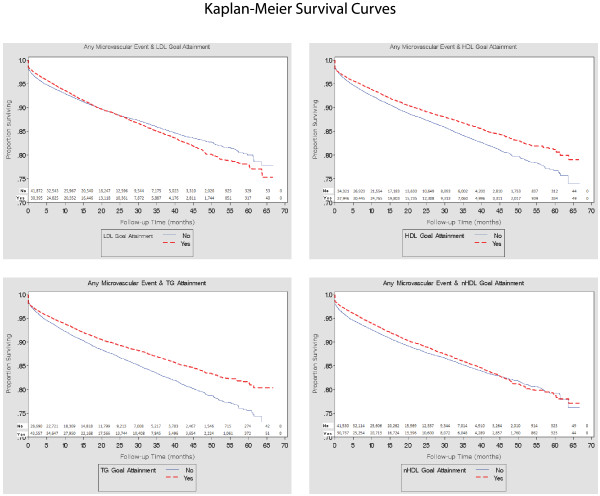
Lipid Subfractions and any Microvascular Event.

The MVC incidence rate for patients who attained their LDL-C goals was 5.17 per 1000 patient-months versus 5.24 for those who did not attain their LDL-C goals during follow up. A unit increase in LDL-C was not associated with a significant risk increase (RR 1.0, [95 % CI, 1–1.001], *P* = .3513). KM curves showed no significant difference in risk for any MVC event and LDL-C goal attainment (RR 1.011, [95 % CI, 0.965-1.059], *P* = .6522; Figure 1). Multivariate Cox regression analysis, however, demonstrated significant associations between LDL-C levels (RR 1.002, [95 % CI, 1.001-1.002], *P* < .0001) and LDL-C goal attainment (RR 0.909, [95 % CI, 0.865-0.955], *P* = .0001) and MVC risk (Table 3).

**Table 3:** Cox Proportional Hazard Models: HDL-C, LDL-C, TG and non-HDL-C and the First Microvascular Event

**Primary Risk Factors of Interest**^**1**^	**Lipid Panel**^**2**^			**Lipid Goal Attainment**^**2**^		
	**HR**^**3**^	**95 % CI**	***P*-value**	**HR**^**3**^	**95 % CI**	***P *-value**
**HDL-C**	0.995	0.993 - 0.997	<.0001	0.895	0.852 - 0.941	<.0001
**LDL-C**	1.002	1.001 - 1.002	<.0001	0.909	0.865 - 0.955	.0001
**TG**	1.001	1.001 - 1.001	<.0001	0.849	0.808 - 0.892	<.0001
**non-HDL-C**	1.003	1.003 - 1.004	<.0001	0.8270	0.788 - 0.868	<.0001

^1^ Four Cox proportional hazard models were run, two including HDL-C, LDL-C, and TG (lipid values and their goal attainment); the others with non-HDL-C lipid values and goal attainment as the primary risk factor of interest.

^2^ The lipid panel values are derived from the last lab assessment results prior to the first microvascular (either retinopathy/nephropathy/neuropathy) event or censorship.

^3^ The HRs were obtained after controlling for the demographics (age, gender and state of residence), other index characteristics (health plan and physician type), pre-index health conditions (hypertension, obesity, heart failure, metabolic syndrome, renal disease, liver disease, ischemic heart disease, peripheral vascular disease, and depression), proportion of days covered during the follow up by the antihypertensives (ACEs, ARBs), and proportion of days covered by lipid altering therapies (statins [low, medium or high], niacin, ezetimibe, or fibrates [fenofibrates or genfibrozil]) between the last lipid panel measurement and event/censorship.

HR: hazard ratio; CI: confidence interval; LDL-C: low-density lipoprotein cholesterol; HDL-C: high-density lipoprotein cholesterol; TG: triglycerides.

#### HDL-C

The MVC incidence rate was 4.73 per 1000 patient-months for patients at their HDL-C goal versus 5.76 per 1000 patient-months who were not. Each unit increase in HDL-C was associated with 1 % decrease in event risk (RR 0.992, [95 % CI, 0.99-0.994], *P* < .0001). Patients who attained HDL-C goals had 17 % lower risk of any MVC event versus those who did not (RR 0.83, [95 % CI, 0.793-0.869], *P* < .0001; Figure 1). Adjusted analysis showed that a one unit increase in HDL-C levels or HDL-C goal attainment reduced MVC risk by 0.5 % (RR 0.995, [95 % CI, 0.993-0.997]; *P* < .0001) and 10.5 % (RR 0.895, [95 % CI, 0.852-0.941]; *P* < .0001), respectively.

#### TG

Patients achieving TG goal had an MVC incidence rate of 4.71 per 1000 patient-months versus those who did not (5.97). A unit increase in TG levels resulted in a 0.1 % greater risk of a MVC event (RR 1.001, [95 % CI, 1.001-1.001], *P* < .0001). Patients at TG goal were 11.3 % less likely to have a microvascular complication versus those not attaining target TG levels (RR 0.787, [95 % CI, 0.751-0.824], *P* < .0001; Figure 1). Multivariate regression analysis also indicated significant associations between TG levels (RR 1.001, [95 % CI, 1.001-1.001], *P* < .0001) and TG goal achievement (RR 0.849, [95 % CI, 0.808-0.892], *P* < .0001) and MVC risk.

#### Non-HDL-C

For patients achieving non-HDL-C goal, the MVC incidence rate was 4.83 versus 5.52 per 1000 patient-months for those not at goal. For each unit increase in non-HDL-C, the increase in MVC event risk was 0.3 % (RR 1.003, [95 % CI, 1.002-1.003], *P* < .0001). Patients who attained non-HDL-C goal had 10 % lower risk for a microvascular event compared with patients who did not (RR 0.90, [95 % CI, 0.859-0.943], *P* < .0001; Figure 1). Adjusted Cox results indicated that non-HDL-C levels (RR 1.003, [95 % CI, 1.003-1.004], *P* < .0001) and non-HDL-C goal (RR 0.827, [95 % CI, 0.788-0.868], *P* < .0001) were significantly associated with lowered MVC event risks.

### Sensitivity analysis

The reported associations for TG and HDL lipid subfractions remained free from any collinearity bias based on the minor (<2) VIF and condition index values observed for the lipid-subfractions in question. This indicates that the associations between TG/HDL and MVCs were not affected by the presence of other lipid fractions in the same model. Irrespective of LDL-C treatment goal achievement, significant independent reductions in MVC risk were found for those who attained HDL-C, TG, HDL-C and TG and non-HDL-C goals versus those who did not achieve these goals (Table 4).

**Table 4:** Sensitivity Analysis: Incidence of MVCs and HDL-C, LDL-C, TG and non-HDL-C Stratified Across LDL-C Goal Attainment

**LDL goal attained**	**HR (95 % C.I)**			
	**HDL goal (yes vs. no)**	**TG goal (yes vs. no)**	**HDL + TG goals (yes vs. no)**	**non-HDL goal (yes vs. no)**
Yes	0.804 (0.75 - 0.862)	0.788 (0.735 - 0.845)	0.758 (0.705 - 0.816)	0.785 (0.717 - 0.86)
No	0.853 (0.802 - 0.907)	0.785 (0.738 - 0.835)	0.787 (0.738 - 0.84)	0.787 (0.711 - 0.871)

HR: hazard ratio; CI: confidence interval; LDL-C: low-density lipoprotein cholesterol; HDL-C: high-density lipoprotein cholesterol; TG: triglycerides.

## Discussion

Consistent with the available evidence on diabetic complications [2,3,7,9-11,17,18], this study found significant independent associations between HDL-C, LDL-C, TG, and non-HDL-C and risks for MVC among patients with T2DM in a real-world managed care population. Although management of LDL-C is the primary goal of therapy, our findings suggest further benefit may be obtained by expanding treatment goals to include modification of other lipid subfractions in addition to LDL-C. These associations persisted after controlling for numerous covariates, including comorbidities and concomitant medications.

A 1 mg/dL increase in HDL-C levels was associated with a significant decrease (0.5 %) in the risk of MVCs. Furthermore, patients who achieved HDL-C goals during follow-up also reduced their risk for experiencing MVCs by 10.5 %. These findings suggest that targeting HDL-C in addition to LDL-C as recommended by NCEP ATP III may have benefits beyond CHD risk reduction [12]. In the case of TGs, a significant increase in risk (0.1 %) for a MVC event was observed for a 1 mg/dL increase in the serum levels of TG. Further underscoring the risks associated with suboptimal TG goals [2,13,19,20], the study found TG goal attainment lowered the risk of MVC by 15.1 %. The relationship of elevated TG and/or reduced HDL-C levels with MVCs was recently demonstrated by Zoppini et al., who showed a high TG/HDL-C ratio approximately doubled the risk for MVC over a 5-year period in 979 Caucasian patients with T2DM [21]. In a prospective observational study of 6,499 patients with diabetes and dyslipidemia, Teramoto et al. confirmed the importance of controlling TG and HDL-C in conjunction with HbA1C levels in patients with T2DM [22]. Taken together, these studies highlight the importance of targeting atherogenic dyslipidemia as a means to reduce MVCs in patients with T2DM. Our results further extend these findings by emphasizing the need to treat HDL-C and triglycerides to ADA defined targets in order to beneficially impact risk for both macrovascular and microvascular events.

Continuous LDL-C levels and achievement of LDL-C goals were positively and negatively associated with MVC event risk, respectively in our study. However, the KM curves between those achieving versus those not achieving LDL-C goals were not statistically different (*P* = .3513), indicating a lack of proportionality of MVC event risk over time. LDL-C reduction does not appear to mitigate risk for MVCs in diabetic patients.

Guidelines recommend non-HDL-C as a secondary target for therapy after risk-stratified LDL-C levels have been reached in patients with baseline TG >200 mg/dL [23]. One of the key results in this study was the 17.3 % reduction in the risk of MVCs for those attaining non-HDL-C goals compared to those not reaching the goals. Non-HDL-C, defined as TC minus HDL-C, is a sensitive surrogate measure of total atherogenic lipoprotein burden in serum and is a useful index for predicting the risk of CVD, especially among patients with diabetes since it circumvents the reliability issues associated with using LDL-C in a diabetic population [24]. These findings hint at a potential role for non-HDL-C in predicting risk for microangiopathy in T2DM patients since it singularly combines the impact of total cholesterol and its various atherogenic lipoproteins in place of LDL-C. Furthermore, evidence exists that after achieving LDL-C goal levels, non-attainment of non-HDL-C target goals among T2DM patients is markedly associated with residual risk and dyslipidemia [25].

Overall, the current study highlights the possible benefits of treating any or all components of the lipid beyond LDL-C alone, in order to most optimally reduce morbidity and mortality in diabetic patients. The sensitivity analyses further confirmed the hypothesis that independently achieving HDL-C, TG, HDL-C and TG and non-HDL-C goals can lead to significant reductions in MVC risk irrespective of LDL-C goal attainment. This suggests that attainment of lipid goals other than LDL-C can translate into incremental reductions in risk of MVCs. Furthermore, this study lays the groundwork for conducting additional research to identify the best predictive models for estimating MVC risk (e.g., correlating Log (TG)/HDL-C with risk for MVC) [26]. More importantly, these study findings suggest that a gap exists between the current practice recommendations and the appropriate management of MVC risk among T2DM patients.

Post hoc analyses of multiple clinical trials suggest that fenofibrate therapy impacts risk for MVC. The Fenofibrate Intervention and Event Lowering in Diabetes (FIELD) study found that patients receiving fenofibrate treatment had a reduced risk of a first and follow-up laser intervention for proliferative retinopathy, lower extremity amputation, and progression to albuminaria and nephropathy versus those on placebo [27-29]. Similarly, the Action to Control Cardiovascular Risk in Diabetes (ACCORD) group of studies evaluating the effect of intensive glycemic control and combination therapy for dyslipidemia found significantly reduced progression rates of diabetic retinopathy for patients who received fenofibrate versus those who received placebo [30]. The Diabetes Atherosclerosis Intervention Study (DAIS) reported reduced progression of albumin excretion for fenofibrate treatment versus placebo [31]. While the confounding effect arising out of fenofibrate usage was controlled for in the present analysis, our results underscore the relative importance of the other components of the standard lipid profile besides LDL-C to microangiopathy risk. They also emphasize the importance of appropriately managing HDL-C, TG, and, non-HDL-C levels through therapeutic lifestyle changes and appropriate pharmacotherapy.

With the increasing prevalence of T2DM adding to the clinical and overall healthcare burdens of patients and society, there is an urgent need for optimal and timely disease management. In the absence of guidelines for the management of lipid subfraction levels to reduce the risk of diabetic microangiopathy, the control of these modifiable risk factors may lead to reducing or delaying the incidence of T2DM-related MVCs [29]. These findings suggest that there may be a need to simultaneously target improvements in multiple lipid subfractions beyond LDL-C, and that pursuing overall lipid subfraction improvements may lead to benefits beyond macrovascular risk reduction for patients with T2DM.

## Limitations

Claims-based studies are subject to important limitations. The coding of claims is susceptible to lack of specificity, miscoding, improper sequencing, and clerical errors. The study may be hindered by incomplete data in terms of insufficient longitudinal capture of relevant claims (e.g. lack of capture of pre-existing MVCs prior to the start of the patient's health plan eligibility) and/or capture of information that is unobservable in an administrative claims database (e.g. use of over-the-counter medications). Unobservable factors that may influence outcomes are generally omitted when using claims data and in the case of this study, the presence of such unobservable factors are assumed to be occur equally between comparison groups. Important contributory factors of T2DM, such as genetics or alcohol or estrogen use, could not be captured in these data [20]. Because the data used in this study pertain to largely working-age subjects within a managed care population, the findings may not be readily replicable or generalizable to the entire T2DM patient population.

The mean follow-up period in our study was less than 2 years, which may not have been of sufficient duration to determine the onset of MVCs attributable to lipid subfractions. However, because this was an observational claims study, requiring a fixed follow-up period of 4 to 5 years for these patients would have forced survival and subsequently introduced bias. As such, follow-up duration was accounted for as a variable in the survival analysis for risk estimation of MVCs. These inherent study design limitations render the findings hypothesis-generating and need to be confirmed in a prospective, randomized clinical trial.

## Conclusion

This study demonstrates significant independent associations among HDL-C, TG and non-HDL-C with risk for microvascular events following the diagnosis of T2DM. Attaining ADA goals for non-HDL-C, HDL-C, and TG levels were significantly associated with a reduced risk of MVCs. There was no clear association, however, between the attainment of the ADA goal for LDL-C and microvascular event risk. These findings suggest that to reduce risk for both macrovascular and microvascular events among patients with T2DM, all components of the lipid profile should be treated to ADA-specified target levels. These findings need to be validated in a prospective, randomized clinical trial.

## Abbreviations

T2DM = Type 2 Diabetes Mellitus; ADA = American Diabetes Association; AHA = American Heart Association; MVC = Microvascular Complication; LDL-C = Low-Density Lipoprotein Cholesterol; TG = Triglycerides; HDL-C = High-Density Lipoprotein Cholesterol; HIRD^SM^ = HealthCore Integrated Research Database^SM^; HIPAA = Health Insurance Portability and Accountability Act; ICD-9-CM = International Classification of Diseases Ninth Revision, Clinical Modification; CPT = Current Procedural Terminology; eGFR = Glomerular Filtration Rate; MDRD = Modification of Diet and Renal Disease; CHD = Cardiovascular Heart Disease; NCEP ATP III = National Cholesterol Education Program Adult Treatment Panel III; ACE = Angiotensin-Converting Enzyme; ARB = Angiotensin Receptor Blockers; PDC = Proportion of Days Covered; KM = Kaplan-Meier; RR = Relative Risk; VIF = Inflation Factor; FIELD = Fenofibrate Intervention and Event Lowering in Diabetes; ACCORD = Action to Control Cardiovascular Risk in Diabetes; DAIS = Diabetes Atherosclerosis Intervention Study; HMO = Health Maintenance Organization; PPO = Patient Preferred Organization; POS = Point of Service; FFS = Fee-For-Service; DCI = Deyo-Charlson Comorbidity Index; SD = Standard Deviation; CI = Confidence Interval; HR = Hazard Ratio.

## Competing interests

Dr. Toth has disclosed that he serves on the Speakers Bureau for Abbott Laboratories, Amylin, AstraZeneca ; GSK, Kowa, and Merck & Co. He is a consultant for Abbott laboratories, Aegerion, Amgen, Amylin, AstraZeneca Pharmaceuticals, Kowa, and Merck. The other authors disclosed that they have no competing interests.

## Authors' contributions

PPT led the study, collaborated in its development and design and reviewed the results and manuscript throughout. RJS conceptualized the study, and reviewed and manuscript the results at all stages. DK conceptualized the study, and collaborated with the team on results evaluation and review. RAQ developed the study design and protocol, supervised the statistical analysis and reviewed the results and manuscript throughout. SRP assisted in protocol development, performed statistical analysis, reported and reviewed the results throughout the study and reviewed and edited the manuscript. MJC reviewed the study design and results at various stages of the process. All authors read and approved the final manuscript.
